# Disease-associated QT-shortage versus quinine associated QT-prolongation: age dependent ECG-effects in Ghanaian children with severe malaria

**DOI:** 10.1186/1475-2875-13-219

**Published:** 2014-06-05

**Authors:** Louise Roggelin, Daniel Pelletier, Josephine N Hill, Torsten Feldt, Steffi Hoffmann, Daniel Ansong, Justice Sylverken, Jürgen Burhenne, Johanna Fischer-Herr, Parisa Mehrfar, Christian Thiel, Gerd D Burchard, Samuel B Nguah, Jakob P Cramer

**Affiliations:** 1 Section Tropical Medicine, Department of Internal Medicine, University Center Hamburg-Eppendorf, Hamburg, Germany; 2 Department Clinical Research, Bernhard Nocht Institute for Tropical Medicine, Hamburg, Germany; 3 Clinic for Gastroenterology, Hepatology and Infectious Disease, University Hospital Düsseldorf, Hamburg, Germany; 4 Pediatric Emergency Unit, Department of Child Health, Komfo Anokye Teaching Hospital, Kumasi, Ghana; 5 School of Medical Sciences, Kwame Nkrumah University of Science and Technology, Kumasi, Ghana; 6 Clinical Pharmacology and Pharmacoepidemiology, Heidelberg University Hospital, Kumasi, Ghana; 7 Paediatric Cardiology, University Center Hamburg-Eppendorf, Kumasi, Ghana

**Keywords:** Malaria, Heart, Children, ECG, QT interval, Quinine, Chloroquine, Wernicke formula

## Abstract

**Background:**

While several anti-malarials are known to affect the electric conduction system of the heart, less is known on the direct effects of *Plasmodium falciparum* infection. Some earlier studies point to a direct impact of *Plasmodium falciparum* infection on the electric conduction system of the heart. The aim of this study was to analyse infection- and drug-induced effects on the electric conduction system.

**Methods:**

Children aged 12 months to 108 months with severe malaria were included in Kumasi, Ghana. In addition to basic demographic, clinical, biochemical and parasitological, biochemical data were measured data upon hospitalization (day 0) and 12-lead electrocardiograms were recorded before (day 0) and after (day 1) initiation of quinine therapy as well as after 42 (±3) days.

**Results:**

A total of 180 children were included. Most children were tachycardic on day 0 but heart rate declined on day 1 and during follow up. The corrected QT intervals were longest on day 1 and shortest on day 0. Comparison of QT intervals with day 42 (healthy status) after stratification for age demonstrated that in the youngest (<24 months) this was mainly due to a QT shortage on day 0 while a QT prolongation on day 1 was most pronounced in the oldest (≥48 months). Nearly one third of the participating children had measurable 4-aminoquinoline levels upon admission, but no direct effect on the corrected QT intervals could be shown.

**Conclusion:**

Severe *P. falciparum* infection itself can provoke changes in the electrophysiology of the heart, independent of anti-malarial therapy. Especially in young - thus non immune - children the effect of acute disease associated pre-treatment QT-shortage is more pronounced than quinine associated QT-prolongation after therapy. Nevertheless, neither malaria nor anti-malarial induced effects on the electrophysiology of the heart were associated with clinically relevant arrhythmias in the present study population.

## Background

The systematic assessment of the effect of severe *Plasmodium falciparum* infection on the electric conduction system of the heart is incomplete. Like other febrile diseases, malaria increases the sympathetic tone in patients, leading to an acceleration of the electric conduction and repolarization of the heart, which can be shown as shortening of the QT intervals in electrocardiographic recordings [1]. However, some studies point to a direct impact of *P. falciparum* infection on the electric conduction system of the heart, which belongs to one of the most severely parasitized organs in humans [2]. Von Seidlein *et al*., for example, found a correlation between parasitaemia and corrected QT prolongation in Gambian children with uncomplicated falciparum malaria [3]. Another study on 161 patients with *P. falciparum* malaria, found abnormal ECG findings in 14.3 per cent of all patients, including T-segment or T-wave alterations in 15 patients and delayed conduction in eight patients [4]. Other studies and case reports point to a rare occurrence of adverse cardiac events during the course of severe malaria infection [5-7].

In addition to potential malaria-related effects, several drugs used for the treatment of *P. falciparum* malaria, such as quinine and halofantrine, are associated with an alteration of the electrophysiology of the heart, in particular provoking QT prolongation [8,9]. This iatrogenic effect has to be accounted for in order to separate malarial and chemotherapeutic effects on the electric conduction system of the heart in severe malaria. As arrhythmias are seldom recognized in children with severe malaria, it is unclear whether or not electrocardiographic alterations are of relevant clinical significance.

This study was designed to measure intra-individual ECG changes at three major points: Day 0 representing malaria without drug effects, day 1 representing malaria and drug effects as well as day 42 representing the healthy status without malaria and anti-malarial drugs. In addition, potential influence of pre-hospitalization medications, age as well as signs and symptoms defining severe malaria on cardiac electrophysiology were assessed.

## Methods

The study was conducted at the Paediatric Emergency Unit of the Komfo Anokye Teaching Hosptial (KATH) in Kumasi, Ghana, between March 2009 and October 2009. After written consent was obtained by parents or legal guardians children aged 12 months to 108 months with signs of severe malaria and a positive rapid diagnostic test (OptiMAL®) were screened. Severe malaria was defined as presence of asexual blood stage parasites, symptoms and conditions according to WHO criteria [10] and by the absence of alternative causes for febrile illness. Metabolic acidosis was defined as base excess < −8.

On admission (day 0) the patients were clinically examined and parameters, such as blood pressure, heart rate, respiratory rate as well as body temperature were recorded and signs of severe malaria were ascertained. Patients were treated with parenteral quinine according to national guidelines (20 mg/kg loading dose followed by 10 mg/kg twice daily) for seven days or until they were able to receive orally administrated artemether-lumefantrine. Patients were followed-up on days 1 and 42 (±3).

A standard 12-lead electrocardiogram (ECG) was conducted on day 0 before the start of quinine therapy, on day 1 after treatment initiation and on day 42. ECGs were recorded at a chart rate of 50 mm/sec. All ECG results were printed, scanned and reviewed using the software GraphicConverter X (Version 6.7.3). An electronic ruler was used to measure RR, PQ, QRS, and QT. To minimize variability due to lead selection, QT-intervals in lead II and V5 were measured and the longer interval was selected for further analysis [11,12]. U-waves were not included in the measurement of the T wave [13]. The QT interval was corrected for heart rate using two different formula, (i) the Bazett formula [14], labelled cQT; and (ii) the Wernicke formula [15], labelled hQT. For subsequent statistical analysis, the formula developed by Wernicke *et al.* was chosen as it more effectively removes the rate dependence of the adjusted QT in children [11]. For comparability with other studies, selected data was also presented according to the Bazett formula.

Blood samples were taken on day 0 and on day 42. Plasmodium falciparum malaria was ascertained from Giemsa-stained thick and thin films. Blood chemistry parameters were measured by the I-STAT® analysing system including but not limited to creatinine, glucose, pH, lactate and base excess. Concentrations of 4-aminoquinoline drugs (chloroquine, N-desethyl-chloroquine, amiodiaquine, N-desethyl-amiodiaqine) were determined from urine samples obtained on day 0 using high-performance liquid chromatography (HPLC) with ultra-violet (UV) detection. The method has already been published with some minor deviations [16]. In brief, isocratic HPLC was performed using 87% TEMED-buffer (tetramethylethylenediamine, 10 mM adjusted to pH 3.5 by phosphoric acid) and 13% acetonitrile as eluent on a Phenomenex Luna C18 column. The flow rate was 1 ml/min and the column was heated to 40°C. The injection volume was 50 μL and the analytes were detected at the UV wavelength of 340 nm. Urine samples were diluted with HPLC eluent (20 μL urine + 1,480 μL eluent containing 4-((7-chloro-4-quinolinyl)amino)-1-pentanol, an amodiaquine analogue, as internal standard) and analyzed by HPLC. Calibrations in urine were done in the range of 3.0–250 μg/mL for all four analytes. The lower limit of quantification was 3.0 μg/mL. The method was validated according to the FDA Guidelines and all accuracy and precision data was within the FDA accepted range of +/−15%.

The study was approved by the ethics committee of the Kwane Nkrumah University of Science and Technology (KNUST), Kumasi. Statistics software SPSS 18.0 was used to analyse data. Statistical significance was defined a p < 0.05. Dependent t-test (pair-wise analysis) was used for comparing findings between day 0, day 1 and day 42. Independent t-test was used to analyse differences in ECG parameters in subgroups.

## Results

### Baseline characteristics

Severe malaria was ascertained and complete day 0-data sets were available for 180 (82%) of 220 initially screened children, 103 (57%) were males. The median age was 36 months (range 12 to 108 months), 125 of the 180 (70%) children were available for follow up ECG assessment on day 42. Criteria for severe malaria were prostration (158 cases), multiple convulsions (94 cases), hyperlactataemia (63 cases), jaundice (55 cases), metabolic acidosis (55 cases), hyperparasitaemia (53 cases), coma (50 cases), severe anaemia (41 cases), haemoglubinuria (36 cases), respiratory distress (22), circular collapse (14), hypoglycaemia (10 cases), renal impairment (3 cases) and abnormal bleeding (1 case). Five children (3%) died. Demographic, clinical and biochemical characteristics are shown in Table 1.

**Table 1:** Demographic, clinical and biochemical characteristics in children with severe malaria on day 0, day 1 and day 42

**Characteristics**	**Day 0 n = 180**	**Day 42 n = 132**	**P value day 42:0**
Age (months, median (± interquartile range))	36 (±26.5)	-	-
Male gender (%)	103 (57)	-	-
Temperature (°C)	37.4 (±1.2)	36.2 (±0.6)	<0.001
Parasite density (per μl)	33.804 (±15.6)	0.6 (±9.0)	<0.001
Respiratory rate (per min)	45.5 (±13.8)	31.5 (±1.2)	<0.001
pH	7.42 (±0.1)	7.42 (±0.1)	0.2
Leukocytes (per μl)	12.3 (±8.5)	9.1 (±3.8)	<0.001
Lactate (mmol/l)	5.2 (±4.2)	2.2 (±1.2)	<0.001
Glucose (mg/dl)	107.4 (±49.1)	94.2 (±18.3)	<0.001
Creatinine (mg/dl)	0.69 (±1.1)	0.36 (±0.1)	0.002

Values are means (±SD) if not indicated otherwise, paired t-test.

### ECG findings

**Figure 1:**
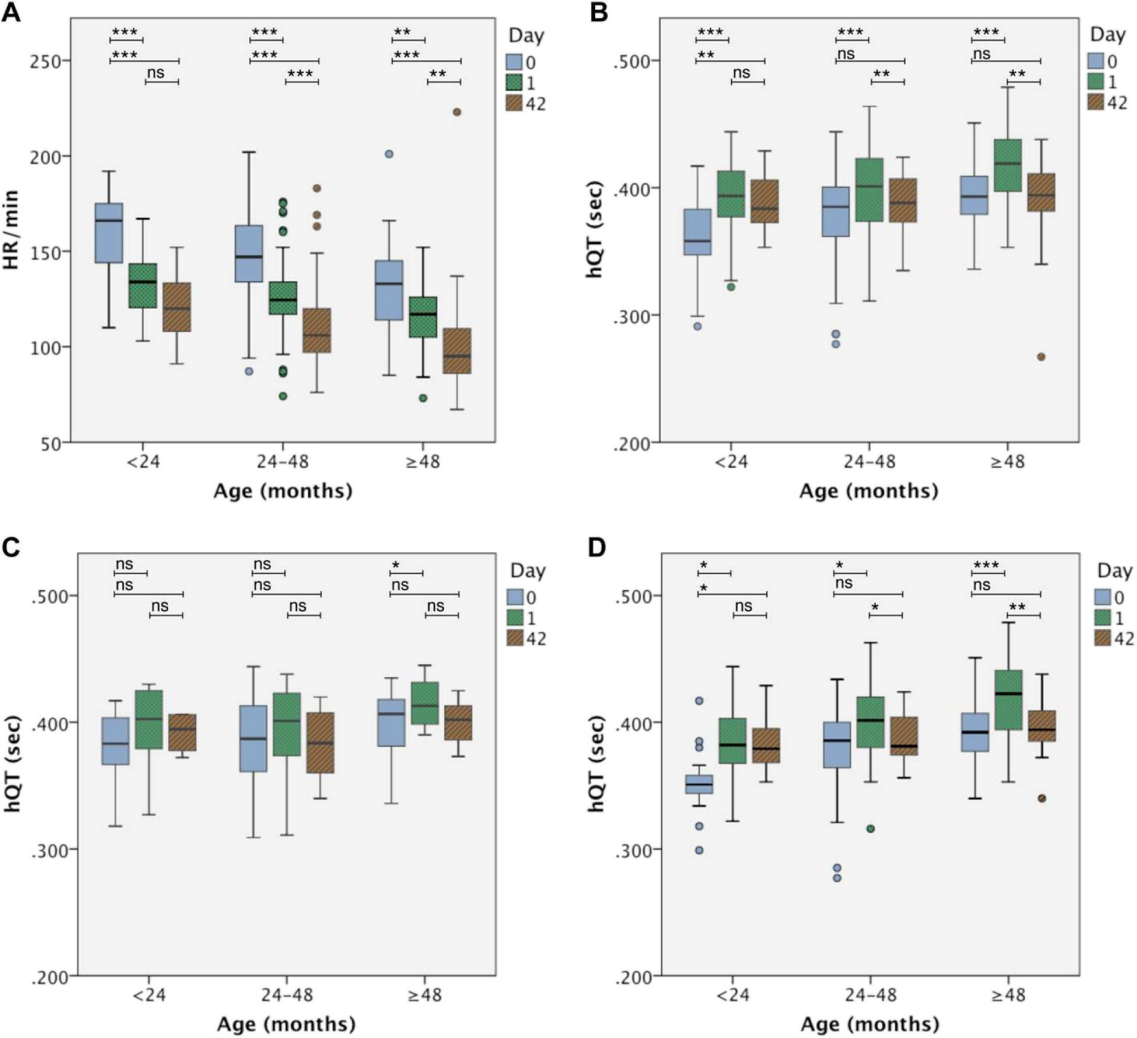
**HR and hQT stratified by age groups (<2 years, 2 to <4 years, ≥ 4).** Heart rate and hQT stratified by age group (<2 years, 2 to <4 years, ≥ 4 years) upon hospitalization in relation to day 1 and day 42: **A** heart rate, **B** hQT, **C** hQT for children *with* positive urine sample of 4-aminoquinoline drugs, **D** hQT for children *without* positive urine sample of 4-aminoquinoline drugs. The boxplots display medians and interquartile ranges. The age groups were compared by t-test: *P < 0.05, **P < 0.005, ***P < 0.001, ns not significant.

Most children had moderate to severe tachycardia on day 0 but heart rates (HR) declined on day 1 and during follow up (Table 2). The corrected overall QT time (hQT) was significantly prolonged on day 1 and shortest on day 0. QRS complex was significantly longer on day 1 compared to day 0, while there was no significant difference between day 0 and 42. The PQ intervals were significantly longer day 1 than on day 0 and during follow up. Comparing hQT and cQT showed overall shorter hQT intervals than cQT intervals (Table 2).For a stratified analysis of the effect of age on ECG parameters, children were divided intro three age groups (<24 months, 24 to <48 months, ≥48 months, Figure 1A and B) on the day of admission. On day 0, HR decreased and hQT increased with age. During the course of disease and follow up, HR decreased over time in all age groups. All age groups showed elevated hQT intervals on day 1 compared to day 0. The hQT intervals in the age group <24 months, were comparable between day 1 and day 42, but significantly shorter on day 0, in the age groups of children 24 months and older, in contrast, the hQT intervals on day 42 were comparable with day 0. The age stratified analysis of the QRS complex revealed that there was only a significant prolongation of QRS on day 1 compared to day 0 in the age group 24 to <48 months (61 vs. 57 ms, p =0.002).

**Table 2:** Electrocardiographic findings on day 0, 1 and 42

**Electrocardiographic findigs**	**Day 0 n = 164**	**Day 1 n = 164**	**Day 42 n = 126**	**P value day 1:0**	**P value day 42:0**	**P value day 42:1**
HR (per minute)	143 (±27)	123 (±23)	106 (±30)	<0.001	<0.001	<0.001
PQ (ms)	121 (±19)	136 (±18)	131 (±20)	<0.001	<0.001	<0.001
QRS (ms)	58 (±12)	63 (±23)	57 (±11)	0.016	0.535	0.048
hQT (ms, Wernicke)	381 (±32)	405 (±32)	390 (±25)	<0.001	0.021	0.001
cQT (ms, Bazett)	422 (±32)	442 (±33)	419 (±27)	<0.001	0.090	<0.001

Values are means (±SD) if not indicated otherwise, paired t-test.

Of the 153 cases in which urine concentrations of 4-aminoquinoline drugs were measured on the day of admission 50 (33%) were positive for one or more than one of these drugs. (N-desethyl)-chloroquine was detectable in 28 of the 50 cases (56%), (N-desethyl-) amiodiaquine was detectable in 37 of the 50 cases (74%), thus 15 cases (30%) were positive for (N-desethyl-) chloroquine and (N-desethyl-) amiodiaquine.

HR was significantly slower in children who had taken 4-aminoquinolines prior to admission compared to those who had not (136 vs. 145, p = 0.048). There was no significant difference in the mean of hQT between children with or without 4-aminoquinoline levels (Table 3).

**Table 3:** hQT time on day 0, 1 and 42 for children with and without positive urine sample of 4-aminoquinoline drugs

**hQT (ms)**	**Day 0**	**Day 1**	**Day 42**	**P value**^**1) **^**day 1:0**	**P value**^**1) **^**day 42:0**	**P value**^**1) **^**day 42:1**
4-aminoquinoline drugs negative	379 (±30) (n = 91)	408 (±32) (n = 91)	392 (±26) (n = 71)	<0.001	0.018	<0.001
4-aminoquinoline drugs positive	386 (±32) (n = 50)	401 (±32) (n = 50)	390 (±25) (n = 36)	<0.001	0.452	0.04
P value^2)^ negative: positive	0.093	0.262	0.299			

Values are means (±SD) if not indicated otherwise. There was no difference in age between both groups.

^1)^Paired t-test.

^2)^Unpaired t-test.

The effect of the pre-hospitalization intake of 4-aminoquinolines was further stratified on hQT according to age (Figure 1C and D). In the subgroup of 4-aminoquinoline naïve children, the hQT intervals were longer on day 1 compared to day 0 in all age groups. As described above hQT was comparable on day 1 and day 42, but significantly shorter on day 0 in the youngest, while in the age groups of children 24 months and older hQT on day 42 was comparable to day 0 (Figure 1D). In the subgroup of children treated with 4-aminoquinolines prior to admission, there was still a trend of longer hQT on day 1 but there was only a significant difference in hQT on day 0 compared to day 1 in the oldest age group (Figure 1C). Nevertheless, there was no significant difference in the mean of hQT intervals between children who had taken 4-aminoquinolines prior to admission compared to those who had not in any age group on day 0 (<2 years: 380 ms *versus* 353 ms p = 0.051, 2 to <4 years: 352 ms *vs* 345 ms p = 0.808, ≥4 years 399 ms *vs* 381 ms p = 0.374).

To further understand the influence of *P. falciparum* infection on the conduction system of the heart, the impact of severe malaria defining conditions on heart rate and hQT intervals upon hospitalization was analysed (Table 4): HR was significantly elevated in children with metabolic acidosis, hyperlactataemia (>5 mmol/l) as well as hyperparasitaemia compared to children without these characteristics. Children with renal impairment had significantly lower heart rates than those without. A significantly shorter hQT was shown in patients with metabolic acidosis and with conditions related to metabolic acidosis such as hyperlactataemia and respiratory distress. Forty-three of 63 patients (68%) with hyperlactataemia and 18 of 22 (88%) patients with respiratory distress also had metabolic acidosis. The hQT was also significantly shorter in patients with prostration. Patients with jaundice and those with haemoglobinuria had a significantly prolonged hQT (Table 4). There was no influence of hypoglycaemia or hyperparasitaemina on hQT intervals (Table 4).

**Table 4:** HR and hQT stratified according to severe malaria-defining sings and symptoms and selected additional parameters on day 0

**Signs day 0**	**No. (%)**	**HR (per min)**			**hQT (ms, Wernicke)**		
		**With sign**	**Without sign**	**P value**	**With sign**	**Without sign**	**P value**
**Clinical signs**							
Coma	48/177 (27)	144 (±25)	142 (±28)	0.688	381 (±34)	381 (±30)	0.996
Prostration	155/177 (88)	142 (±28)	142 (±23)	0.983	379 (±34)	396 (±28)	0.025
Multiple convulsions (per 24 h)	92/177 (52)	141 (±26)	143 (±28)	0.617	381 (±37)	381 (±30)	0.967
Respiratory Distress	22/177 (12)	152 (±40)	141 (±25)	0.069	360 (±40)	384 (±31)	0.002
Jaundice	53/177 (30)	137 (±23)	144 (±28)	0.086	391 (±34)	377 (±30)	0.010
Haemoglubinuria	36/175 (21)	136 (±25)	144 (±28)	0.147	391 (±34)	379 (±32)	0.046
Circularly collapse	14/178 (8)	147 (±21)	143 (±25)	0.506	384 (±34)	381 (±33)	0.700
**Laboratory signs**							
Hypoglycaemia	10/177 (5)	140 (±51)	142 (±25)	0.805	375 (±20)	381 (±34)	0.595
Metabolic acidosis (BE < −8)	55/171 (32)	152 (±32)	137 (±23)	<0.001	367 (±32)	388 (±32)	<0.001
Severe anaemia (Hb < 5 g/dl)	41/164 (25)	149 (±35)	140 (±24)	0.052	383 (±33)	373 (±33)	0.089
Hyperparasitaemia	53/177 (30)	151 (±21)	138 (±28)	0.002	374 (±29)	384 (±35)	0.086
Hyperlactataemia (>5 mmol/l)	63/172 (37)	155 (±28)	134 (±24)	<0.001	367 (±38)	389 (±27)	<0.001
Renal impairment (creatinine >3 mg/dl)	3/163 (2)	106 (±31)	142 (±24)	0.016	411 (±34)	382 (±32)	0.128
**Additional parameters**							
Male gender	102/177 (58)	141 (±30)	144 (±22)	0.548	379 (±33)	384 (±33)	0.247
Capillary refill time ≥ 3 s	17/177 (10)	135 (±42)	143 (±25)	0.255	382 (±34)	380 (±33)	0.863

Values are means (±SD) if not indicated otherwise, unpaired t-test.

No clinically significant arrhythmias were observed in any of the subjects during the study assessment. However, no continuous ECG-monitoring was performed. No comparisons were made between children with fatal and nonfatal severe malaria because of the low number of fatalities.

## Discussion

Both, changes in the heart rate (tachycardia, bradykardia) as well as cardiac arrhythmias have the potential to influences cardiocirculatory function and, therefore, to gain clinical relevance. Children with severe malaria were tachycardic on the day of admission but the heart rate declined with the onset of anti-malarial therapy to a level considered as normal on day 42 reflecting the state of acute febrile illness and subsequent recovery.

Corrected QT intervals (hQT) were shorter before therapy than during therapy and also shorter before therapy compared to after cure. The hQT prolongation on day 1 was probably at least partially related to quinine [1]. The data suggest, however, that at least in the youngest children hQT-reduction related to acute disease is more important in contributing to the observed increase in QT-time day 0 to day 1 than a potentially drug-related QT-prolongation on day 1. As day 42 reflects the normal state in healthy children, the hQT intervals on admission are not only shorter compared to the situation under quinine therapy but also shorter in the treatment-naïve ill child than in the cured child in this age group. This QT-shortage on day 0 is likely related to the effects of acute stress and increased sympathetic tone on day 0 and relative improvement of the physical condition one day after the start of the treatment [1]. These findings are in line with Adjei *et al.*, who also saw a relative shortening of pre-treatment QT intervals in children with uncomplicated malaria [17]. The QRS intervals were longest on day 1, which can at least partially be explained by quinine effects. Quinine slows depolarization by slowing rapid upstroke of cardiac action potential by blocking sodium inward current (I_NA_) and thereby widening QRS complex, an effect earlier described in other studies [18,19] and also seen in this study.

Stratification revealed some age-specific effects on both heart rate and repolarization. HR was highest in the youngest and lowest in the oldest children on day 0, 1 and 42, respectively, resembling the physiological decline of heart rates with age. The age stratified hQT intervals on day 42, reflecting the healthy status, were comparable in all age groups confirming the validity of the applied correcting formula for the QT interval. Nevertheless, there are differences in the characteristics of the hQT intervals between the different age groups during acute disease: In the groups 24 to <48 months of age and >48 months of age there was a significant prolongation of hQT on day 1 after the start of quinine therapy in comparison to day 0 as well as to day 42. In the group of children younger than 24 months of age, on the other hand, there was no significant difference in hQT intervals between day 1 and day 42 (Figure 1B). Therefore, the QT-shortage during acute disease seems the most prominent in the youngest children while quinine related QT-prolongation becomes increasingly more important with age. Increased sympathetic tone due to stress, anxiety and discomfort have been described to increase heart rate and conduction, resulting in QT shortening [1]. The iatrogenic effects of the anti-malarial are difficult to separate from the effect of the underlying disease but the data suggests a more pronounced effect of the disease itself rather than the anti-malarial on myocardial electrophysiology itself. Furthermore, a prolongation of the QRS intervals on day 1 – likely representing the direct effect of quinine therapy – could only be shown in age group 24 to <48 months. Van Hensbrook *et al.* observed that QRS prolongation after the onset of quinine therapy was most pronounced in children younger than 24 months [18]. The data from this study contradict these findings by revealing an acute disease related QT-shortage rather than a drug related QT prolongation at least in the youngest children.

As children younger than 24 months still lack partial immunity to malaria, the age-dependent differences in ECG findings might be due to immunity-dependent differences in pathophysiology of parasite-host-drug interactions. An earlier study demonstrated an age dependent effect of plasma nitric oxide (NO) on parasite density in Ghanaian children with severe malaria with the highest NO levels in the youngest children [20]. NO is known to play a key role in regulating cardiac function [21,22].

A relatively high percentage of children had taken 4-aminoquinoline drugs prior to admission. Despite the change of national policies in 2009 an unexpected high portion was still treated with chloroquine. Chloroquine is considered to potentially prolong QT interval [23,24], but there is no evidence for clinical cardiotoxicity after oral administration of anti-malarial treatment doses of chloroquine [1]. In this study there was no significant difference between hQT on day 0 in children who had been treated with chloroquine or amiodiaquine prior to admission to those who had not been treated – neither for the whole study group nor for age stratified subgroups. Nevertheless, there are some interesting findings when comparing the age-stratified data of hQT for the different groups. QT-shortage during acute disease in particular in the youngest and QT-prolongation during quinine treatment in the elderly were more pronounced in 4-aminoquinoline-naïve than in pre-treated children.

Of the symptoms and characteristics of severe malaria, some conditions seem to influence the electrophysiology of the heart prior to quinine therapy while others do not. Metabolic acidosis - present in one-third of all children – as well as respiratory distress and hyperlactataemia were associated with a shortening of hQT intervals. Respiratory distress as a clinical sign of metabolic acidosis was present in most of the acidotic children and both respiratory distress and acidosis are considered as independent predictors of fatal outcome in falciparum malaria [25,26]. How these changes in the electrophysiology of the heart also influence cardiac function is difficult to say. While an impaired cardiac output in acidotic children could not be shown in the same study population [27] others indeed identified impaired cardiac function in relation to acidosis [27,28]. Children with hyperlactataemia and metabolic acidosis also showed increased HR. These findings can probably be seen as the overall activation of autonomic nervous system in severely ill children. While earlier studies showed an impact of hyperparasitaemia [3] and hypoglycaemia [29] on corrected QT intervals, the data from this study does not support these findings. Further, lower heart rate in children with renal impairment could be shown but this particular subgroup was too small to allow a general conclusion from the data.

The study had a number of limitations: a problem involved the determination of the corrected QT intervals: Many formulas have been proposed to correct QT intervals for the heart rate [14,15,30,31] but none is completely satisfying. In this study population of severely ill children, most heart rates measured were over 100 beats per minute. As Bazett’s formula overcorrects the QT interval in high heart rates it was considered as inadequate for this study population and it was decided to use the Wernicke formula developed for children and adolescents [15]. As normal limits for the paediatric ECG, including QT intervals, have been described mostly for Bazett`s formula [32], Bazett corrected QT intervals (cQT) were still included in some of the analyses to facilitate comparison to findings in other studies. Comparing hQT and cQT showed overall shorter hQT intervals than cQT intervals and thereby demonstrating the overcorrection of the Bazett’s formula. Even if the Wernicke seems to be more accurate for this study population one still has to keep in mind, that effects of age and gender on the QT interval are described [15,33] and how error-prone every calculation is as even time of day, fever, autonomic function and changes in posture can influence the QT interval [34-37]. Thus, the optimal formula might be hard to find.

## Conclusions

In conclusion, the findings suggest that severe malaria itself is associated with changes in the electrophysiology of the heart, independent of anti-malarial therapy. Especially in young - thus non-immune – children acute malaria-associated QT-shortage seemed to be more prominent than anti-malarial-associated QT-prolongation. Nevertheless, it remains unclear whether these ECG-changes are of high clinical relevance as cardiac arrhythmias and subsequent circulatory dysfunction does not seem to be a major contributor to unfavourable outcomes in severe malaria.

## Abbreviations

cQT: Bazett corrected QT interval; ECG: Electrocardiogram; hQT: Wernicke corrected QT interval; HR: Heart rate; KATH: Komfo Anokye Teaching Hosptial; KNUST: Kwame Nkrumah University of Science and Technology; Min: Minute; ms: Milliseconds; NO: Nitric oxide; SD: Standard deviation.

## Competing interests

The authors declare that they have no competing interests.

## Author’s contribution

DP and SH participated in patient recruitment and data collection. LR and DP performed the statistical analyses and drafted the manuscript. JNH participated in statistical analysis. JPC and SBN conceived of the study and participated in its design and coordination and helped to draft the manuscript. JB performed the urine sample analyses. TF, DA, JS, JFH, PF, CT and GDB significantly contributed to the protocol development and/or study implementation. All authors read and approved the final manuscript.
